# Health behaviours during the coronavirus disease 2019 pandemic: implications for obesity

**DOI:** 10.1017/S1368980020003031

**Published:** 2020-12

**Authors:** Niyati Parekh, Andrea L Deierlein

**Affiliations:** Public Health Nutrition, School of Global Public Health, New York University, 715-719 Broadway, Room 1220, New York, NY 10012, USA

**Keywords:** Obesity, Coronavirus disease 2019, Diet, Physical activity, Health behaviours

## Abstract

**Objective:**

Obesity is a risk factor for severe complications and death from the coronavirus disease 2019 (COVID-19). Public health efforts to control the pandemic may alter health behaviors related to weight gain, inflammation, and poor cardiometabolic health, exacerbating the prevalence of obesity, poor immune health, and chronic diseases.

**Design:**

We reviewed how the pandemic adversely influences many of these behaviors, specifically physical activity, sedentary behaviors, sleep, and dietary intakes, and provided individual level strategies that may be used to mitigate them.

**Results:**

At the community level and higher, public health and health care professionals need to advocate for intervention strategies and policy changes that address these behaviors, such as increasing nutrition assistance programs and creating designated areas for recreation and active transportation, to reduce disparities among vulnerable populations.

**Conclusions:**

The long-lasting impact of the pandemic on health behaviors, and the possibility of a second COVID-19 wave, emphasize the need for creative and evolving, multi-level approaches to assist individuals in adapting their health behaviors to prevent both chronic and infectious diseases.

Obesity is a major public health concern in the USA. There are approximately 42 % of Americans with obesity (defined as a BMI > 30 kg/m^2^), of which 9 % suffer from severe obesity (BMI > 40 kg/m^2^)^(1)^. Emerging evidence suggests that obesity is a strong risk factor for severe complications, hospitalisation and death from the coronavirus disease 2019 (COVID-19)^(2)^. In New York City, compared with adults (aged < 60 years) with a BMI < 30 kg/m^2^, those with a BMI 30–34 kg/m^2^ and those with a BMI > 35 kg/m^2^ were 1·8 (95 % CI 1·2, 2·7) times and 3·6 (95 % CI 2·5, 5·3) times more likely to be admitted to acute and critical care, respectively^(3)^. Although the specific biological mechanisms continue to be elucidated, inflammation and immune dysregulation are central to the aetiology of COVID-19, attacking the lungs and vasculature system and progressing to the heart, kidneys and other organs throughout the body^(2,4)^. Individuals with obesity may be particularly susceptible to COVID-19 infection due to the range of comorbidities associated with excess adiposity, including hyperglycaemia, hypertension, inflammation and impaired respiratory function^(5)^.

While social distancing measures are necessary to control the pandemic, they will also have unintentional consequences that may worsen the obesity epidemic and its related comorbidities in the USA. Sheltering-in-place has significantly altered health behaviours and the food environment by limiting opportunities for daily physical activities, encouraging screen time and sedentary behaviours, disturbing sleep and promoting consumption of ultra-processed foods and alcohol. All of these behaviours may contribute to weight gain and the development of cardiometabolic diseases, such as diabetes, hypertension and CVD. During this time, public health professionals are faced with the dual challenge of continuing to promote obesity prevention strategies, while supporting COVID-19 containment efforts. Herein, we discuss modifiable behavioural risk factors for weight gain that have been affected by the pandemic: physical activity, sedentary behaviours, sleep and diet (Fig. 1). We provide strategies to improve them, which can be incorporated into public health messaging, interventions and tele-medicine during this period.

**Fig. 1:**
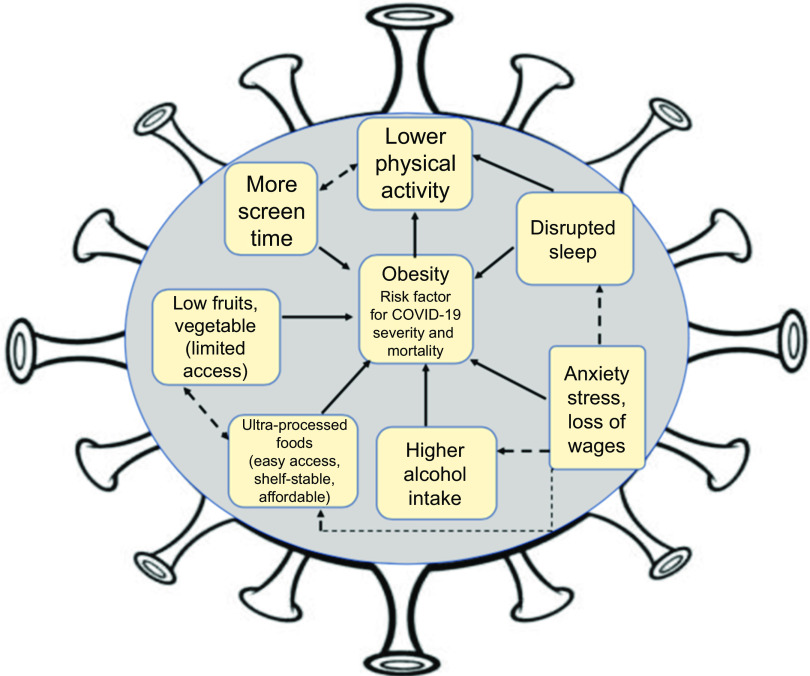
Interrelationships of behavioural risk factors for weight gain that have been affected by the COVID pandemic; the confluence of these behavioural changes is hypothesised to exacerbate the national prevalence of obesity that is a threat for disease severity and mortality

## Physical activity

The majority of American adults do not meet the national recommendations of at least 150 min of moderate physical activity or 75 min of vigorous physical activity (or an equivalent combination) per week^(6)^. Physical activity is associated with a wide range of meaningful health benefits. Individuals who engage in regular physical activity are more likely to have a healthy weight status; reduced risks of cardiometabolic diseases, some cancers and osteoporosis; improved cognition; and shorter periods of depression and anxiety^(7)^. Physical activity is also critical for improving quality of life among individuals with chronic conditions and disabilities. Social distancing and lockdown measures have diminished opportunities to participate in several domains of physical activity, particularly those related to recreation, transportation and work. Recreational sources of activity, such as health clubs, gyms, pools, and indoor and outdoor sports facilities (e.g., tennis and basketball courts), have limited access and even after re-opening many individuals may be reluctant to use them. Daily activities associated with transportation and work, for example, walking a child to the bus stop, running errands or climbing a flight of stairs at the office, have also been drastically reduced. These short bouts of activity throughout the day are important contributors to daily energy expenditure and weight gain prevention and allow for breaks in sedentary behaviours. During this time, efforts should be made to schedule movement throughout the day, particularly for individuals who do not have a regular exercise routine. Walking is an excellent low-impact exercise that can improve cardiovascular fitness and increase energy expenditure, even in short 10-min increments^(8)^. Caregiving and household activities, such as playing with children, cleaning and gardening, may also be used to off-set lost daily activities from other domains. Additionally, at-home exercise classes and programmes are available for free or low cost on many social media platforms (e.g., Instagram, YouTube and Facebook) for various types of exercises and skill levels. Information on these resources and other physical activity strategies can be tailored and distributed to target populations.

## Sedentary behaviours

The average American adult spends 7·2–9·5 h/d engaged in sedentary behaviours, such as sitting while working, reading, watching television and using computers, smartphones or other devices^(9)^. Although the overall evidence for an association between sedentary behaviours and obesity in adults is inconsistent, sitting for extended time periods is associated with greater waist circumference and higher blood levels of TAG, glucose and insulin, which are biomarkers of poor cardiometabolic health^(10)^. The types of sedentary behaviours that individuals engage in are also important. Among all of the sedentary behaviours, television watching likely has the greatest influence on weight gain due to the obesogenic behaviours that accompany it^(9)^. Television watching is an environmental stimulus that increases food intakes, independent of hunger-satiety signals or food palatability; therefore, consumption of meals and energy-dense snack foods in front of the television may result in excess energy, fat and sugar intakes^(11)^. Television watching also exposes individuals to advertisements for unhealthy foods and beverages, which may further encourage their consumption^(12)^. For the majority of individuals, stay-at-home measures have increased sedentary time, especially among apartment dwellers. Physical activity breaks throughout sedentary time can reduce sitting-related health risks and are associated with lower BMI and improved cardiometabolic health biomarkers^(13)^. Individuals can also keep meals and snacking separate from all work-, school- and leisure-related sedentary behaviours to reduce opportunities for overeating and junk food consumption.

## Sleep

Modern society has resulted in an increased prevalence of deficient sleep health, which encompasses inadequate sleep duration, poor sleep quality and sleep disorders. The average American adult suffers from deficient sleep due to sleeping less than the recommended 7–8 h/night, having a job that requires shift work and/or having a sleep disorder (e.g., insomnia, sleep apnoea)^(14)^. Deficient sleep is associated with increased risks of diabetes, hypertension, CVD and obesity^(14)^. Experimental studies demonstrate that restricted or impaired sleep reduces glucose tolerance and insulin sensitivity and alters appetite-regulating hormones, resulting in decreased satiety and increased feelings of reward and pleasure in response to food stimuli^(15)^. Individuals with short or disrupted sleep report greater energy intakes, which may be attributed to more frequent meal occasions, larger portion sizes and preference for energy-dense foods that are high in fat and carbohydrates^(16)^. Moreover, energy expenditure from physical activities is often reduced among people with inadequate sleep^(16)^. The pandemic may disrupt and shorten sleep in several ways, including altering usual bed times (e.g., going to bed later), increasing screen time and intensifying anxiety and stress levels. Individuals may be able to overcome some of this disruption by maintaining good sleep hygiene practices, such as setting a consistent sleep/wake routine, extending sleep duration to meet recommended amounts and avoiding or reducing blue light exposure from screen use (using blue light blocking glasses or software) around bedtime, since blue light may interfere with melatonin levels and stimulate brain activity^(17)^. Limiting late-night snacking and alcohol consumption and achieving recommended amounts of daily physical activity also help to regulate sleep.

## Diet

The external and household food environments are strongly correlated with individual diet quality and health. Greater access to and purchases of fruits, vegetables and whole grains are associated with higher nutrient intakes, improved immune function and reduced chronic illness, while greater access to and purchases of ultra-processed foods are associated with nutrient deficiencies and chronic disease development^(18)^. Ultra-processed foods are defined as industrially manufactured, ready-to-eat or ready-to-heat formulations, which contain little to no whole, fresh foods. Prior to the pandemic, ultra-processed foods constituted the majority of energies purchased by US households and were the main source of total and added sugars, Na and fats. Although some ultra-processed foods may provide vitamins and other essential nutrients (e.g., vitamin C, *n*-3 fatty acids and folic acid), the majority of these foods contain preservatives and additives (e.g., Na, trans fats, high fructose maize syrup, artificial colourings, nitrites and sulphites), as well as neo-formed contaminants and chemicals^(19,20)^. These ingredients are hypothesised to influence cardiometabolic disease development through several mechanisms, including dysregulating blood lipid, glucose and hormone concentrations; altering gut microbiota; increasing body fat stores; and generating oxidative stress and inflammation^(18,21)^. Additionally, ultra-processed foods are hypothesised to promote poor dietary habits, such as snacking and overeating, due to their convenience, omnipresence, low cost and large portion sizes^(18)^.

The pandemic has drastically changed the food environment. Record high unemployment rates compounded by interruptions in the food supply chain due to worker shortages, heightened safety inspections and delays in the transportation and delivery of fresh foods have left consumers with no choice but to consume what they can afford and access at their local food stores. Lockdown measures have also reduced the frequency of grocery shopping, further decreasing the ability to purchase perishable fresh foods, particularly produce. Rates of household food insecurity are mounting and early reports from grocery retail stores demonstrate historically high sales of shelf-stable and ultra-processed foods, such as boxed macaroni and cheese and snack foods, as well as alcohol^(22,23)^. Aside from issues surrounding substance abuse and mental health, alcohol consumption is associated with stimulating appetite, overeating and weight gain. Although achieving optimal dietary quality is challenging during this time, individuals can make several efforts to increase their intakes of nutrient-dense foods. Grocery store purchases should focus on frozen, canned (low Na varieties) or dried plant-based items, like whole grains (e.g., brown rice, whole grain maize meal, whole wheat pasta and oats), pulses (legumes and beans), vegetables and fruits, as well as fresh produce that does not quickly perish, such as apples, pears, cabbage, carrots, squashes, sweet potatoes and beets. Gardening, even in window sills and balconies, may further help to encourage consumption of vegetables, fruits and fresh herbs. Stay-at-home measures have also increased reliance on cooking and baking, which provides the opportunity to make more healthful versions of store-bought processed foods. For example, soups and stews, pizza, breads and cookies may all be home made with whole grains, vegetables and low sugar and salt content. Beverage consumption should focus on varieties of water (e.g., plain, seltzer and fruit infused), while avoiding all sugar-sweetened beverages. The US Dietary Guidelines recommend that if alcohol is consumed then it should be done in moderation, defined as up to one drink for women and two drinks for men per day^(24)^. For individuals who consume alcohol, daily drinking should be limited and should not be higher than pre-pandemic intakes, which may suggest that alcohol is being used as a coping mechanism for social isolation, boredom and other stressors.

## Conclusions

Historically, obesity has been of public health concern due to its strong associations with chronic disease morbidity and mortality. The COVID-19 pandemic has highlighted that obesity greatly increases susceptibility to complications and mortality from infectious diseases as well. Public health measures to control the pandemic may alter health behaviours related to weight gain, inflammation and poor cardiometabolic health, exacerbating the prevalence of obesity, poor immune health and chronic diseases in the USA and other countries with developed economies. However, for many of these behaviours, specifically physical activity, sedentary behaviours, sleep and dietary intakes, the influence of the pandemic can be mitigated. Table 1 summarises individual-level practices related to each behaviour that may be promoted during this time. At the community level and higher, public health and health care professionals need to advocate for intervention strategies and policy changes that address these behaviours. For example, expanding community nutrition services and government food assistance programmes to reduce disparities among vulnerable populations or increasing designated areas for recreation and active transportation, such as green spaces, street traffic closures and protected bike lanes to provide safe spaces for individuals to exercise, walk, run and bike while maintaining social distance, particularly in urban areas. The long-lasting impact of the pandemic on health behaviours and the possibility of a second COVID-19 wave emphasise the need for creative and evolving, multi-level approaches to assist individuals in adapting their health behaviours to improve immune function and prevent both chronic and infectious diseases.

**Table 1:** Individual-level practice recommendations related to physical activity, sedentary behaviours, sleep and diet during the COVID pandemic

Aim to accumulate at least 30 min/d or 150 min/week of moderate physical activity; engage in at activities within the home or in socially distanced outdoor activities such as walking, jogging or biking Incorporate movement breaks during sedentary work periods or during extended periods of screen time Limit late-night snacking and avoid eating in the absence of hunger Limit consumption of packaged salty and sweet foods and sugar-sweetened beverages Consume more plant-based foods, specifically whole grains, vegetables, fruits, lean proteins and dairy. Substitute with low-sugar, low-salt frozen or canned items if fresh produce is unavailable Cook healthy meals at home Maintain good sleep hygiene practices; aim for at least 7 h of sleep every night, avoid screens, bright lights, and caffeinated and alcoholic drinks before bed Consume alcohol in moderation or do not drink at all Cope with stress by doing breathing exercises, yoga, meditation, engaging in regular activity and ensuring sufficient sleep
